# Montreal Veterinary Medical Association

**Published:** 1895-11

**Authors:** Harri H. Dell

**Affiliations:** Secretary-Treasurer


### MONTREAL VETERINARY MEDICAL ASSOCIATION.

THE twenty-first annual opening meeting was held in the library of the Faculty of Comparative Medicine on Thursday evening, October 10, 1895, with the President, Dr. Adami, in the chair. There was a good attendance of members and candidates for admission.

Minutes of last meeting were read and approved.

The special business of the meeting was the election of officers for the ensuing year.

The honorary President, Dr. D. McEachran, took the chair, and the ballot resulted in the election of the following: President, Dr. M. C. Baker; First Vice-President, Dr. N. D. Gunn; Second Vice-President, Mr. E. C. Thurston; Secretary-Treasurer, Harri D. Dell; Librarian, Mr.J. A. Ness.

Fourteen new members were added to the roll, and Dr. Martin, Professor of Pathology, was elected to honorary membership.

Dr. Adami referred to the admirable condition of the library, which was largely due to the zeal and energy of Mr. Thurston, the retiring Librarian.

A reporting committee was appointed, consisting of Messrs. F. W. Kee and S. C. Richards, to act with the Secretary in communicating reports of the meetings to the various professional journals.

Dr. Baker thanked the association for the honorable position to which they had called him, and outlined the work for the year.

Dr. Adami assured the members of his continued interest in the work of the association.

Messrs. Greer and McCarrey were instructed to procure material for experimental purposes, and the essayists for the next meeting appointed, after which the meeting adjourned.

HARRI H. DELL,
Secretary-Treasurer.

